# P2RP: a web-based framework for the identification and analysis of regulatory proteins in prokaryotic genomes

**DOI:** 10.1186/1471-2164-14-269

**Published:** 2013-04-20

**Authors:** Mohamed Barakat, Philippe Ortet, David E Whitworth

**Affiliations:** 1 CEA, DSV, IBEB, SBVME, LEMiRE, Saint-Paul-lez-Durance, F-13108, France; 2 CNRS, UMR 7265, Saint-Paul-lez-Durance, F-13108, France; 3 Aix-Marseille Université, Saint-Paul-lez-Durance, F-13108, France; 4 Institute of Biological, Environmental and Rural Sciences, Aberystwyth University, Ceredigion, UK

## Abstract

**Background:**

Regulatory proteins (RPs) such as transcription factors (TFs) and two-component system (TCS) proteins control how prokaryotic cells respond to changes in their external and/or internal state. Identification and annotation of TFs and TCSs is non-trivial, and between-genome comparisons are often confounded by different standards in annotation. There is a need for user-friendly, fast and convenient tools to allow researchers to overcome the inherent variability in annotation between genome sequences.

**Results:**

We have developed the web-server P2RP (Predicted Prokaryotic Regulatory Proteins), which enables users to identify and annotate TFs and TCS proteins within their sequences of interest. Users can input amino acid or genomic DNA sequences, and predicted proteins therein are scanned for the possession of DNA-binding domains and/or TCS domains. RPs identified in this manner are categorised into families, unambiguously annotated, and a detailed description of their features generated, using an integrated software pipeline. P2RP results can then be outputted in user-specified formats.

**Conclusion:**

Biologists have an increasing need for fast and intuitively usable tools, which is why P2RP has been developed as an interactive system. As well as assisting experimental biologists to interrogate novel sequence data, it is hoped that P2RP will be built into genome annotation pipelines and re-annotation processes, to increase the consistency of RP annotation in public genomic sequences. P2RP is the first publicly available tool for predicting and analysing RP proteins in users’ sequences. The server is freely available and can be accessed along with documentation at http://www.p2rp.org.

## Background

In prokaryotes biological processes tend to be regulated at the level of transcription, with subsets of genes/operons being up/down-regulated by specific DNA-binding proteins known as transcription factors (TFs). TFs can be divided into a few major categories, including sigma factors (SFs), one-component systems (OCSs) and response regulators (RRs), and the DNA-binding activity of these proteins is often regulated. SFs are the specificity-conferring sub-units of RNA polymerase holoenzymes, and they direct the transcription machinery towards particular promoter sequences [1]. The activity of SFs is often regulated by accessory proteins such as anti-SFs, which bind to and inhibit specific SFs. In addition to DNA-binding domains, OCSs possess sensory domains, which modulate DNA-binding activity according to the presence/absence of a particular stimulus [2]. Finally, the DNA-binding activity of RRs is regulated by the phosphorylation-state of their receiver domains, which can be phosphorylated by partner receptor kinase proteins called histidine kinases (HKs). Together HKs and their partner RRs (including non-DNA-binding RRs), form two-component systems (TCSs), which are the dominant phosphorylation-dependent signal transduction pathways of prokaryotes [3].

A typical prokaryotic genome encodes around 5% TFs [4] and 1.5% TCS proteins [5], and for most regulatory proteins (RPs), multiple homologues are usually found in each genome. Therefore for RPs, sequence similarity does not necessarily imply a similar functional role, and annotation of RPs by sequence similarity has resulted in many erroneous annotations.

Over-specific annotation is a common problem. For example, the *E. coli* PhoB/OmpR family of RRs regulate diverse processes, including potassium homeostasis (KdpE), copper tolerance (CusR) and trimethylamine N-oxide respiration (TorR), in addition to phosphatase expression (PhoB) and osmoregulation (OmpR) [6-10]. However multiple PhoB/OmpR family members in a genome are sometimes ascribed the same role. For example, *Clostridium botulinum B* str. Eklund 17B encodes 28 OmpR family RRs, of which seven are annotated as regulating phosphatase expression, and 11 are annotated as being VanR, which regulates vancamycin resistance [11].

Due to intrinsic problems in defining the physiological function of regulatory proteins by sequence homology, functional annotation by sequence similarity has now largely been superseded by categorisation on the basis of domain architecture [12-14]. In this manner, RPs can be divided into families, and family membership then correlates with mechanism of action rather than biological function. Several on-line databases are now available which provide the results of such classification approaches as applied to whole genomes. For example, P2CS [15], P2TF [4], MiST2 [16] and DBD [17].

However there are remaining problems with RP annotation. Many RPs contain multiple domains, and some domains are found in multiple categories of RP. This has led historically to the mis-annotation of many proteins. For instance, SAB1964 is an RR from *Staphylococcus aureus* RF122, yet it is annotated as a ‘two component sensor protein’, while YPA_3835 is a HK from *Yersinia pestis* Antiqua, which is annotated as an ‘ATPase-like ATP-binding protein’. Currently, 1.5% of all proteins now classified as RRs in the P2CS database [15], were originally described in some way as ‘sensor kinase’ proteins in the annotated genome files as retrieved from Refseq/Genbank. This problem is exacerbated by the current lack of a community-defined consensus set of categorisation criteria, or even a consensus naming system, for multi-domain RPs. However, this has been accomplished for a subset of RPs (RRs), by Galperin [13,14].

Due to their multiplicity within genomes and their multi-domain architectures, RPs are non-trivial to identify and annotate. Currently, the annotation of regulatory genes/proteins in individual genomes and databases is often idiosyncratic, misleading or wrong, confounding between-genome comparisons, and naming conventions are also typically different between genomes/databases. There is consequently a profound need for the adoption of a consistent and harmonised categorisation and annotation system for RPs, which can be applied to any sequence dataset, whether newly derived sequences needing annotation, or previously annotated sequences which might benefit from re-annotation [3].

We have therefore developed P2RP (Predicted Prokaryotic Regulatory Proteins) – primarily to help increase (re-)annotation consistency of RPs in published genomes, and for experimental biologists who wish to investigate regulatory genes in their novel sequence data. P2RP accepts two types of input – DNA and protein sequences. For nucleotide queries there is an initial gene prediction step (using MED-Start) to generate a proteome, although. gbk (GenBank) files can also be inputted. Predicted and supplied proteomes are then screened for the presence of particular TF/TCS domains, and proteins categorised and annotated according to their domain architecture [15,18]. Every user query is given an ID, which allows later retrieval of results, and results of the P2RP process can be viewed as a web server interface page, or downloaded in a variety of user-specified formats. P2RP can be accessed at http://www.p2rp.org and is free and open to all users, with no login requirement.

## Implementation

**Figure 1:**
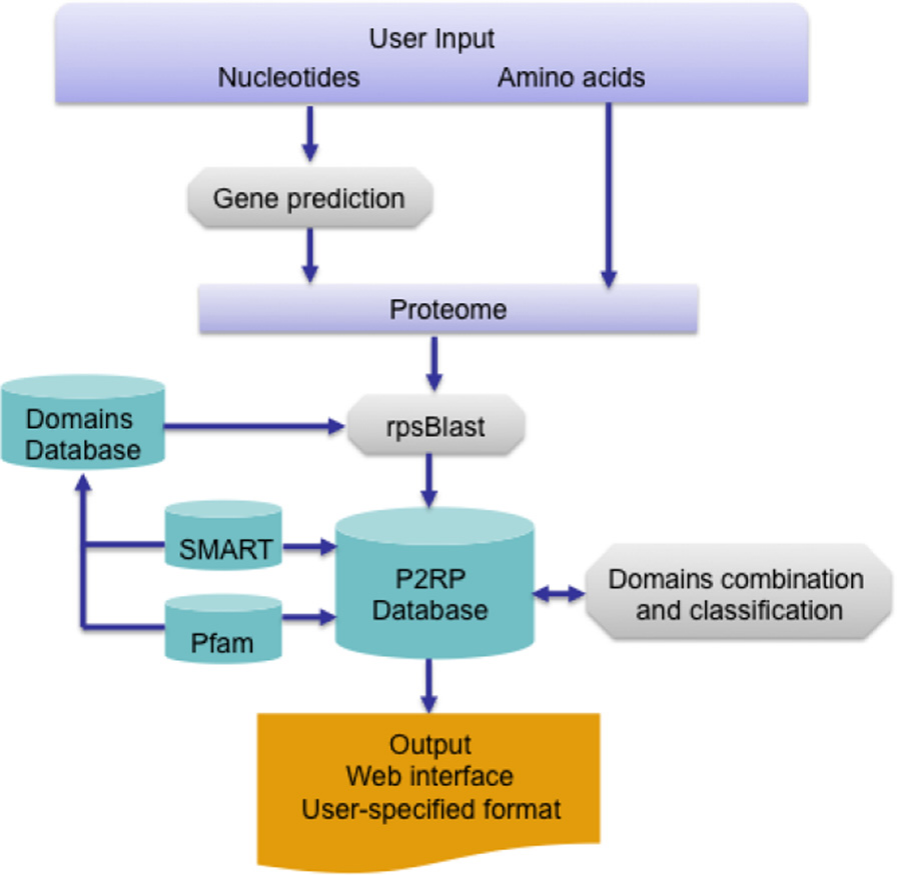
**The P2RP workflow.** If users upload a nucleotide sequence query there is an initial gene-prediction step to yield protein sequences for analysis. Protein sequences (whether predicted by P2RP or inputted by the user), are then screened for the presence of developer-specified sets of conserved domains using SMART and Pfam domain profiles. Proteins with hits to appropriate domain profiles are then used to populate a ‘P2RP Database’. Assignment of P2RP Database proteins to specific categories and sub-categories of RPs is then achieved using ‘Domains combination and classification’ algorithms. The results of the RP analysis can then be viewed as an interactive webpage or exported in a user-defined format.

A graphical representation of the P2RP process is shown in Figure 1. Users can upload sequences into P2RP as.gbk (GenBank) files, or as multiple FASTA files of protein or DNA sequences (for instance whole metagenomes). In the case of nucleotide FASTA files, the sequence is first run through MED-Start [19], a prokaryotic gene-finding algorithm, to define a set of potential gene sequences. The putative genes are then translated to constitute a proteome for RP prediction.

The identification of RP candidates in protein sequences is accomplished by domain analysis of each predicted protein, using RPSBLAST, as previously developed for P2CS and P2TF [4,15,18]. The pool of domains used to search for RP proteins was manually selected from the literature and extracted from within Pfam [20] and SMART [21] databases. P2RP is a set of PHP scripts (PHP: Hypertext Preprocessor, a server-side scripting language designed for Web development), designed to search the numerous combinations of RP modules and to categorize RP proteins into families based on similarity and/or domain architecture. To circumvent the prediction of false-positive RPs, a post-analysis process is implemented. For instance, enzymes erroneously classified as RP proteins and enzymes containing DNA-binding domains (for instance transposases) are discarded or categorized as ‘Other DNA-binding Proteins’ (ODPs) respectively. The secondary structure of RP proteins is computed using the PSIPRED method [22]. The result of the protein structure prediction is presented as a summary of the number of strands and helices and their location on the protein sequence, on which the identified domains are highlighted. Finally, the cellular localization of each TCS protein is determined by the presence or absence of transmembrane (TM) segments, using the HMMTOP predictor [23]. The server time required for execution of these procedures is less than 100 seconds for genomes of up to 4 megabases in size (Intel Xeon 6-Core 2x2.66 GHz).

**Figure 2:**
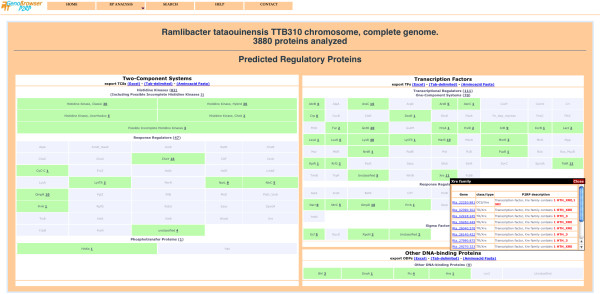
**P2RP results page for a nucleotide format query.** The left half of the screen describes TCS genes, while the right half describes TF genes (including the subset of TCS genes that encode DNA-binding domains). When mousing-over the category ‘Response Regulators’, a gene list popup window shows all ten such genes. In addition to displaying annotation (both categorisation and domain architecture), links are provided to each individual gene page.

**Figure 3:**
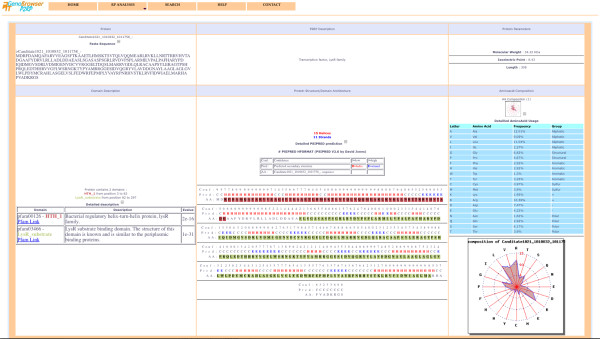
**Individual P2RP gene page.** For each TF/TCS gene a page is available showing the results of a variety of analyses of the predicted gene product, including links to secondary structure prediction, amino acid composition, biochemical parameters and sequence data.

Once processing is complete, the results are summarised and displayed as a web page (Figure 2), which shows global counts of the different categories of RPs and detailed class counts of each category. Each class result provides a detailed gene list, via a popup window when the mouse is passed over active text (Figure 2). For each entry within the gene list, a link takes the user to a new page, which provides detailed annotation for the gene product, including a P2RP annotation, a domain description, secondary structure prediction, amino acid composition, biochemical parameters and sequence data (Figure 3). In addition, for follow-up analysis, external links to other web servers are available, including links from results to the P2CS and P2TF databases of genomic/metagenomic RPs.

P2RP results can be outputted for download in a user-specified format, as an Excel worksheet (see Additional file 1 and Additional file 2 for examples), FASTA file or as tab-delimited text. To keep each user session private, every user query is given an ID, which allows later retrieval of results, using the ‘Search’ menu. The data are stored on the server for one month. For easy navigation, in addition to the homepage, each P2RP page contains a navigation bar that allows users to execute a new RP analysis, to search for a previous job, to access a help page or to contact the authors.

## Results and discussion

### Annotation and classification of RPs

The classification strategy for TCS proteins was based on that implemented by the P2CS database of genomic and metagenomic TCSs [15,18]. Possession of one of an established list of TCS domains defines proteins as being TCS components. Identified TCS proteins are then classified based on domain composition [13], for example the presence of HATPase and HisKA domains leads to classification of a protein as a HK. At this stage proteins are classified as RR, HK or phosphotransfer proteins (which usually shuttle phosphoryl groups between receiver domains).

RRs are then assigned to families according to the nature of any output domain present on the protein, for instance any RRs containing a HTH_8 DNA-binding domain are classified as PrrA family members, while HKs are sub-classified as classic, hybrid, unorthodox, CheA or ‘possible incomplete’ (those lacking an obvious phosphorylatable His residue).

In addition to TCS proteins, P2RP also identifies and categorises DNA-binding proteins (classing them as TFs), if they give hits to a compiled pool of DNA-binding domains from the Pfam and SMART libraries, in a scheme developed initially for P2TF [4]. TFs are then divided into families according to their domain architecture as proposed previously [4,17,24,25], and as implemented in the P2TF database [4]. The P2RP analysis then identifies TF proteins also containing a CheY-like receiver (phosphoacceptor) domains and annotates them as RRs. SFs were divided into 3 sub-groupings; RpoN, RpoD and ECF (extra-cytoplasmic function) SFs. OCSs were defined as proteins that contain sensory ‘input’ and DNA-binding ‘output’ domains but lack histidine kinase and receiver domains characteristic of two-component systems [2]. TFs with only a DNA-binding domain are named transcriptional regulators (TRs). OCSs, TRs, RRs and other non-SF DNA-binding proteins are then divided into 76 families depending upon which domains are present in the proteins. For instance MerR family members contain MerR DNA-binding and B12-binding domains (a protein domain which binds to cobalamin (vitamin B_12_)).

Validation of the categorisation process was achieved by comparing P2RP output with manually curated datasets, as has been described for P2TF and P2CS [4,15,18]. Since P2RP implements the same algorithms developed for P2CS and P2TF, validation statistics (including sensitivity and specificity) for TCS prediction and TF prediction can be obtained from the help pages at http://www.p2cs.org and http://www.p2tf.org respectively. Hundreds of inputs were analysed during testing (amino acid and nucleotide sequences), and beyond the authors, fourteen individuals were involved in the testing and validation (including undergraduate students of Aberystwyth University registered on module BS33120 Molecular Genetics of Microbes).

We have thus developed a high-quality automated analysis system, which builds homogenized genome annotations and increases the consistency of RP prediction in publicly available genomic sequences. As well as assisting experimental biologists, P2RP could be built into genome annotation pipelines, and could thereby generate a significant proportion of all genome annotation. For instance, in a rifamycin SV-producing actinomycete, *Amycolatopsis mediterranei* U32, over 14% of proteins are predicted and annotated as RPs.

## Conclusion

Regulatory processes are fundamental to how microbes alter gene expression in response to environmental changes such as those encountered during infection. Regulation is achieved by RPs such as transcription factors and two-component system proteins. RPs are relatively difficult to annotate because of their multi-domain nature and their paralogy within most prokaryotic genomes. P2RP provides users with the opportunity to investigate and consistently annotate RPs within novel sequence data, or to re-annotate published sequences. It is hoped that this will prove a useful resource to experimental biologists, in addition to increasing consistency in the annotation of RPs in public databases - potentially being used routinely within annotation pipelines.

### Availability and requirements

• **Project name:** P2RP.

• **Project home page:**http://www.p2rp.org.

• **Operating system(s):** Platform independent.

• **Programming language:** PHP, JavaScript.

• **License:** This website is free and open to all users and there is no login requirement.

## Abbreviations

RP: Regulatory protein; TF: Transcription factor; SF: Sigma factor; RR: Response regulator; HK: Histidine kinase; TCS: Two-component system; OCS: One-component system; ODP: Other DNA-binding protein; TM: Transmembrane; ECF: Extracytoplasmic function.

## Competing interests

The authors declare that they have no competing interests.

## Authors’ contributions

MB and PO developed and designed the web-server. DW participated in the improvement of the web-server functionalities. DW drafted the manuscript and MB and PO revised it. All authors have read and approved the final submitted version of this manuscript.

## Supplementary Material

Additional file 1 Nitratifractor salsuginis DSM 16511. Click here for file

Additional file 2 Haloquadratum walsbyi DSM 16790. Click here for file
